# T lymphocyte heterogeneity in NSCLC: implications for biomarker development and therapeutic innovation

**DOI:** 10.3389/fimmu.2025.1604310

**Published:** 2025-05-29

**Authors:** Yu Liu, Denghui Qin, Jiejun Fu

**Affiliations:** ^1^ Center for Translational Medicine, Guangxi Medical University, Nanning, Guangxi, China; ^2^ Key Laboratory of Longevity and Aging-Related Diseases, Ministry of Education, Nanning, Guangxi, China; ^3^ Guangxi Key Laboratory of Brain Science, Guangxi Medical University, Nanning, Guangxi, China

**Keywords:** melanoma, T lymphocyte, CD8 + T cell, PD-1, NRAS mutations, immunotherapy combination therapy, biomarkers

## Abstract

Non-small cell lung cancer (NSCLC) immunotherapy has been revolutionized by immune checkpoint inhibitors (ICIs), yet response heterogeneity persists due to dynamic tumor-immune interactions. This review summarizes recent studies in understanding tumor-infiltrating lymphocyte (TIL) biology, highlighting CD8^+^ cytotoxic T cells and regulatory T cells (Tregs) as pivotal regulators of immune surveillance and suppression. We summarize emerging biomarkers such as TCR clonality, spatial distribution of tumor-infiltrating lymphocytes (TILs), and exhaustion markers including PD-1, TCF1, and TIM-3, which predict immune checkpoint inhibitor (ICI) efficacy beyond PD-L1 expression. This review specifically describes radiotherapy-induced immunogenic remodeling and peripheral T cell dynamics as innovative strategies to monitor immune response and resistance mechanisms. By integrating results from single-cell omics and AI-driven spatial analysis, we propose multidimensional frameworks of TIL in NSCLC to overcome resistance and optimize immunotherapy combinations. These insights collectively advance NSCLC immunotherapy toward precision modulation of the tumor immune microenvironment.

## 1 Introduction

Lung cancer remains the leading cause of malignancy-related mortality worldwide, with non-small cell lung cancer (NSCLC) accounting for over 85% of histological subtypes (1). Although immune checkpoint inhibitors (ICIs) targeting axes such as PD-1/PD-L1 have revolutionized therapeutic paradigms, patient response rates remain constrained by the dynamic heterogeneity of the tumor microenvironment (TME) (2). Studies demonstrate that tumor-infiltrating T lymphocytes (TILs), as core TME components, form dual regulatory networks through subtype distribution and functional states: CD8^+^ T cells mediate tumor cell killing via the perforin-granzyme system (3), while regulatory T cells (Tregs) foster disease progression by establishing an immunosuppressive niche through IL-10/TGF-β (4–6).

The cancer immunoediting framework further reveals that tumor cells drive T cell exhaustion via PD-1, CTLA-4, and Tim-3, characterized by loss of effector function, inhibitory receptor upregulation, and metabolic reprogramming (7–11). Notably, exhausted T cells retain partial clonal expansion potential, offering therapeutic targets for ICI intervention (12–15). Single-cell sequencing identifies PD-1^+^CD8^+^ T cell clonal expansion as a predictive biomarker for ICI efficacy (16), while CD4^+^ T cells synergistically amplify antitumor immunity by regulating dendritic cell antigen presentation (17, 18) and inducing CD8^+^ T cell IFN-γ secretion (17). Recent advances highlight activated CD8^+^ T cells as inducers of tumor ferroptosis (19), though their efficacy is compromised by Treg/CD8^+^ ratio imbalance (12) and spatial distribution heterogeneity (20–23). Current research focuses on deciphering epigenetic remodeling mechanisms underlying T cell exhaustion and integrating single-cell omics with spatial transcriptomics to transcend limitations of traditional biomarkers like PD-L1, thereby propelling NSCLC immunotherapy from empirical practice toward multidimensional precision modulation.

## 2 Multidimensional regulatory networks of tumor-infiltrating lymphocytes in NSCLC

Tumor-infiltrating lymphocytes, as key effectors within the tumor immune microenvironment, play a pivotal role in determining NSCLC prognosis and therapeutic response. TILs are primarily composed of T lymphocytes (24), including CD8^+^ cytotoxic T cells that mediate tumor cell lysis through perforin–granzyme pathways (25, 26), CD4^+^ helper T cells (Th1/Th2) that regulate cellular and humoral immunity via IL-2/IFN-γ and IL-4/IL-5, respectively, and Foxp3^+^ Tregs that exert immunosuppressive effects through IL-10 and TGF-β secretion (27–29). Single-cell sequencing reveals spatial-specific functional specialization in CD4^+^ and CD8^+^ T cells (24), with their equilibrium predicting immunotherapy outcomes. Current biomarkers face limitations: PD-L1, the sole FDA-approved ICI marker, remains limited by tissue heterogeneity and detection variability (30), while threshold ambiguities and translational challenges hinder TMB and ctDNA clinical application (31, 32). Novel strategies leverage baseline TCR diversity (3) and spatial CD8+ TIL patterns, including associated with prognosis in stage I–IIIA NSCLC (33) and tumor nest localization observed in stage IV disease (34). Terminally exhausted CD8^+^ TILs (TIM^-^3^+^PD-1^+^) require CTLA-4 inhibitor-driven Treg depletion combined with PD-1 blockade (4, 35, 36), while epigenetic reversal of exhaustion (16) and adoptive therapy dose-responses (3) offer multidimensional interventions. Future integration of single-cell omics and spatial transcriptomics must map TIL functional subsets and spatial niches, propelling NSCLC immunotherapy toward systems biology-driven precision (37, 38).

### 2.1 The dual roles of tumor-infiltrating T lymphocytes in NSCLC tumorigenesis and progression

Tumor-infiltrating T lymphocytes in NSCLC exhibit dual functions, either suppressing or promoting tumor progression. This dynamic interplay reflects the immune system’s balance between resisting immune evasion and sustaining antitumor responses (39). As primary antitumor effectors, CD8^+^ CTLs eliminate tumor cells via perforin/granzyme-induced membrane disruption, Fas/FasL-mediated apoptosis, and TNF/TNFR signaling amplification (40). CD4^+^ Th1 cells further support this by presenting antigens (MHC-II), secreting IFN-γ and TNF-α to activate antigen-presenting cells and enhance MHC-I expression, and mediating direct tumoricidal effects via FasL (41). Together, these cells coordinate spatiotemporally to form a robust T cell-mediated cytotoxic network. Conversely, Foxp3^+^ Tregs and Th2 cells foster tumor immune evasion. Tregs enhance tumor invasiveness through Foxp3-dependent epigenetic reprogramming (42), secrete IL-10 and TGF-β to inhibit CD8^+^ T cell cytotoxicity (43), and downregulate costimulatory molecules, hindering effective T cell activation. Th2 cells complement this by activating the IL-4/Gata3/STAT6 axis, inducing genes linked to proliferation and metastasis, and promoting integrin-mediated tumor invasion (44). The synergistic action of Tregs and Th2 cells reshapes tumor immunoediting via paracrine cytokine signaling and cell–cell interactions, ultimately undermining immune surveillance and advancing malignancy (**Table 1**).

**Table 1:** Tumor-infiltrating lymphocyte (TIL) subtypes in NSCLC.

TIL Subtype	Functional Role	Key Mechanisms	Prognostic Association
CD8+ Cytotoxic T	Direct tumor lysis via perforin/granzyme; IFN-γ-mediated TME remodeling	Fas/FasL, TNF/TNFR pathways; Induces tumor ferroptosis	High intratumoral density: OS HR=0.52, PFS HR=0.52; Stromal density (stage I-IIIA): HR=0.62
CD4+ Th1	Enhances CTL activation via IL-2/IFN-γ; MHC-II antigen presentation	Synergizes with DCs; FasL-mediated direct cytotoxicity	IFN-γ+IL-17A+ naïve CD4+ T cells predict ICI response (AUC=0.849)
CD4+ Th2	Promotes immune evasion via IL-4/IL-5	STAT6 activation; Integrin-mediated tumor invasiveness	Elevated Th2/Treg ratios correlate with advanced metastasis
Foxp3+ Treg	Immunosuppression via IL-10/TGF-β; Inhibits CD8+ T cell function	Downregulates APC costimulatory molecules; Epigenetic reprogramming via Foxp3	Foxp3+/CD8+ ratio >0.3: HR=2.15 for recurrence; Independent risk factor
Exhausted CD8+	Loss of effector function; Partial clonal expansion potential	PD-1/CTLA-4/TIM-3 upregulation; Metabolic reprogramming	TIM-3+PD-1+ terminal exhaustion requires dual ICI therapy; TCF1+PD-1+ predicts better PFS

### 2.2 Prognostic significance of tumor-infiltrating T lymphocytes in NSCLC

The prognostic value of TILs hinges on subtype-specific distribution, spatial localization, and functional states (45, 46), with CD8^+^ CTLs exhibiting marked prognostic heterogeneity. MANDARANO et al. (47) demonstrated that intratumoral CD8^+^ TILs correlate with favorable outcomes, while a meta-analysis by LI et al. (48) further revealed that high intratumoral CD8^+^ TIL density associates with prolonged overall survival, progression-free survival, and a 4.08-fold increase in objective response rate following immunotherapy, though peripheral blood CD8^+^ T cell levels show no clinical relevance. XIA et al. (49) identified significant enrichment of IFN-γ^+^IL-17A^+^CD4^+^ naïve T cells and PD-1^+^CTLA-4^+^CD4^+^ memory T cells in responders to anti-PD-1 therapy, whereas elevated CTLA-4^+^CD4^+^ memory T cells predict poor prognosis in anti-PD-L1 treatment. Notably, while increased CD8^+^ TIL density paradoxically correlated with reduced 5-year survival, both CD3^+^ TIL abundance and IL-2-high subgroups demonstrated significant survival benefits. This counterintuitive observation suggests that functional activation status may serve as a more reliable prognostic indicator than mere lymphocyte subtype density (50). Mechanistic studies on Foxp3+ Treg-mediated protumor effects (42) reveal their suppression of CD8^+^ CTL cytotoxicity and enhancement of tumor invasiveness, with elevated Foxp3^+^/CD8^+^ and Foxp3^+^/CD4^+^ ratios confirmed as independent risk factors for postoperative recurrence (51). Current heterogeneity in findings likely stems from methodological disparities, sampling sites, and analytical threshold variability, underscoring the urgent need for standardized multidimensional frameworks integrating TIL spatial distribution, clonal diversity, and functional activation to establish reliable prognostic models for NSCLC precision immunotherapy.

### 2.3 The role of tumor-infiltrating T lymphocytes in NSCLC immunotherapy

**Figure 1:**
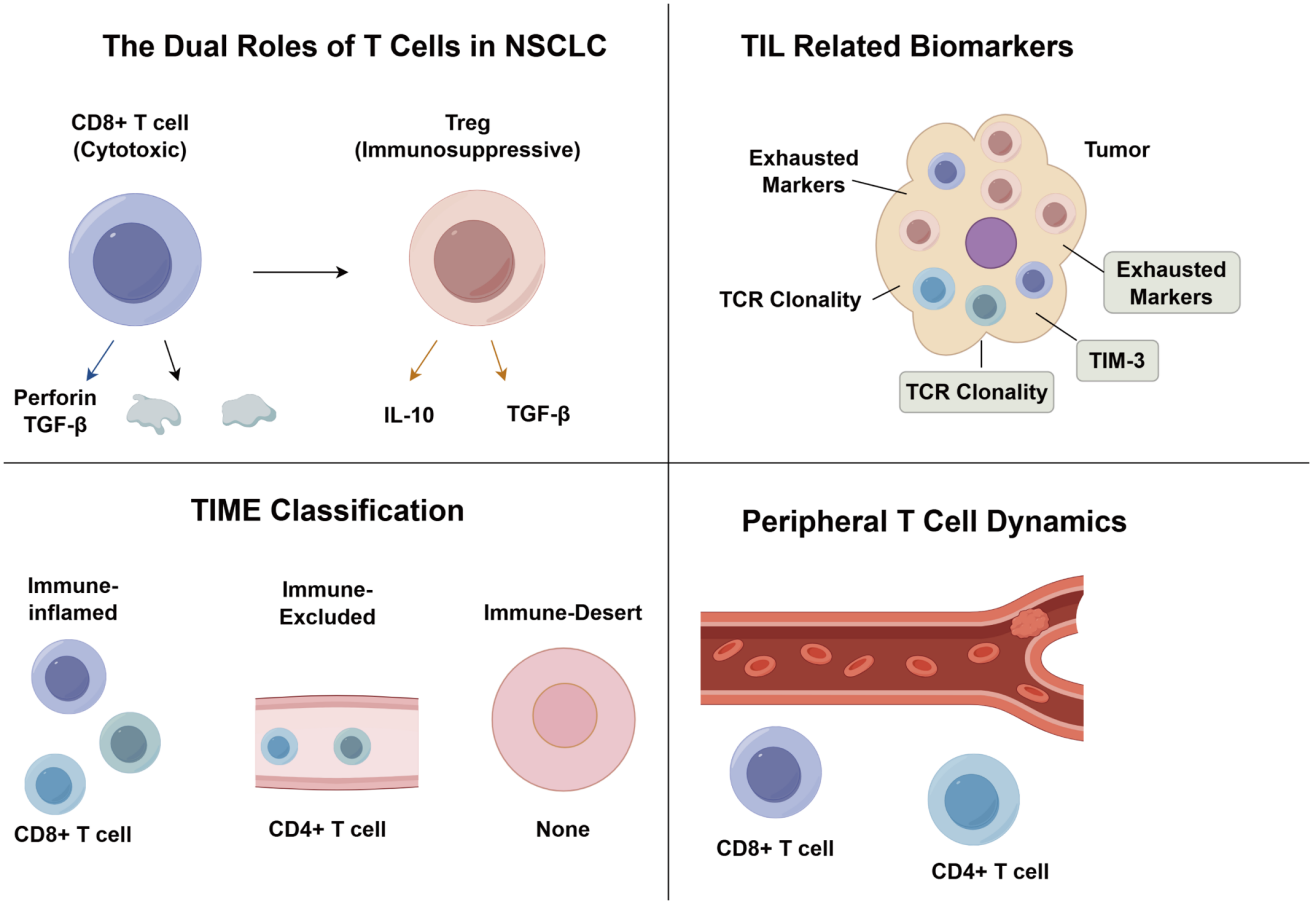
The role of tumor-infiltrating T lymphocytes in NSCLC.

Despite the transformative clinical impact of ICIs in NSCLC, heterogeneous patient responses underscore the urgent need for precision biomarkers. High TIL density is significantly associated with prolonged progression-free and overall survival following immunotherapy (52), underscoring TILs as predictive biomarkers of treatment responsiveness. Immune-inflamed tumors with high TIL infiltration demonstrate superior clinical outcomes compared to immune-desert phenotypes lacking immune cells, while immune-excluded and immune-suppressed subtypes exhibit intermediate responses (53). Multidimensional immunohistochemical analysis by KIM et al. (54) revealed that ICI responders display elevated CD3^+^ and CD8^+^ TIL densities, increased CD8^+^/CD3^+^ ratios (reflecting effector T cell activation), and reduced Foxp3^+^/CD8^+^ ratios (indicative of immunosuppressive microenvironment attenuation). Multivariate regression identified CD3^+^ TIL densityand Foxp3^+^/CD8^+^ ratio as independent predictors of ICI clinical benefit. Notably, EGFR-mutant tumors exhibit markedly diminished CD3^+^ TIL infiltration, providing a microenvironmental basis for their reduced ICI responsiveness. Collectively, these findings advocate for composite predictive models integrating T cell subset spatial distribution (intratumoral vs. stromal) and functional activation/exhaustion markers, thereby advancing precision stratification beyond PD-L1 monotherapy paradigms (**Figure 1**).

## 3 Roles of T cells in defining the NSCLC tumor immune microenvironment

### 3.1 Tumor immune microenvironment classification

The TIME classification system, based on TIL spatial patterns, stratifies tumors into immune-inflamed, immune-excluded, and immune-desert subtypes (55, 56). Immune-inflamed tumors feature dense CD8^+^/CD4^+^ T cell infiltration and PD-1/PD-L1 activation near tumor nests, correlating with better responses to ICIs. In contrast, immune-excluded tumors are characterized by stromal T cell accumulation without infiltration into the tumor parenchyma, while immune-desert tumors are devoid of T cells altogether, both exhibiting limited sensitivity to immune checkpoint inhibitors. To overcome the subjectivity of traditional histopathology, Park et al. (57) developed an AI-based whole-slide imaging (WSI) model to classify TIME phenotypes using H&E slides. This system predicted clinical outcomes, with immune-inflamed tumors showing significantly improved progression-free survival and overall survival versus immune-excluded and immune-desert subtypes. Expanding on this, Teng et al. (58) introduced a four-tier model incorporating PD-L1 status and TIL density: Type I (PD-L1^+^/TIL^+^), Type II (PD-L1^-^/TIL^-^), Type III (PD-L1^+^/TIL^-^), and Type IV (PD-L1^-^/TIL^+^). Shirasawa et al. (59) validated its prognostic value, showing that Type I had the highest response rate and longest median PFS, while Type III reflected resistance due to immune exhaustion. Further refinements include Wu et al’s (60) identification of stage-specific TIME features and transcriptomic model (61), which defines immune-enriched, immune-enriched fibrotic, fibrotic, and depleted TIME subtypes. Notably, immune-excluded subtypes respond best to ICIs, and therapy-induced TIME transitions highlight the plasticity of the immune landscape. Together, these evolving frameworks support multidimensional TIME classification, but require further multicenter validation and mechanistic dissection of treatment-induced remodeling.

### 3.2 T Cell-related biomarkers in TIME

The dual PD-L1 expression on tumor and immune cells confers dynamic biological functions. High infiltration of CD8^+^PD-L1^+^ TILs exhibited hot tumor features but correlated with shorter progression-free survival due to concurrent CD68^+^ macrophage and CD163^+^ M2 polarization fostering an immunosuppressive niche (62). Conversely, in advanced patients receiving PD-1 inhibitors, the high CD8^+^PD-L1^+^ TILs group showed improved objective response rate and PFS via T cell exhaustion reversal. This duality underscores PD-L1’s spatiotemporal regulatory role—exacerbating Treg-mediated suppression in native immunity while serving as a therapeutic vulnerability under ICI intervention—complementing TIME classification theories and offering a novel composite biomarker for precision immunotherapy stratification.

Emerging evidence highlights the clinical significance of T cell exhaustion states in NSCLC. Pre-exhausted TCF1^+^PD-1^+^ populations demonstrate superior prognostic value compared to terminally exhausted TIM-3^+^TIGIT^+^ subsets, as revealed through single-cell sequencing (63). In clinical validation, abundant TCF1^+^PD-1^+^ tumor-infiltrating lymphocytes correlated with sustained treatment benefit in a 116-patient surgical cohort receiving immune checkpoint inhibitors, suggesting these pre-exhausted cells maintain functional memory potential (64). However, CD8^+^PD-1High TILs exhibiting TIM-3/CTLA-4 co-expression along with impaired IFN-γ/TNF production showed opposite associations with reduced disease-free survival (65), a pattern subsequently confirmed in advanced NSCLC cohorts (66). This dichotomy mirrors PD-L1^+^CD8^+^ TIL dualism, advocating dynamic models integrating exhaustion-stage-specific markers. While limited by retrospective designs, these findings highlight multidimensional T cell functional assessment as a breakthrough beyond PD-L1 limitations.

Beyond CD8^+^ T cells, CD4^+^ T cell subsets demonstrate distinct prognostic value in NSCLC through neoantigen recognition and immunomodulatory functions. In advanced NSCLC, elevated FoxP3^+^CD4^+^ TIL infiltration was associated with improved progression-free and overall survival (67), potentially reflecting regulatory T cell-mediated mitigation of T cell exhaustion. Spatial transcriptomic analyses using digital profiling further revealed that CD4^+^ T cell localization within specific immune niches significantly enhanced survival outcomes, with observed synergistic effects from co-localized CD56^+^ NK cells (68). This spatially resolved approach advances beyond conventional immunohistochemistry by precisely mapping topological relationships between CD4^+^ T cells and NK cells, offering three-dimensional insights into tumor immune microenvironment heterogeneity. When integrated with T cell exhaustion profiling, these spatial and functional characterization methods collectively enhance precision immunotherapy strategies.

## 4 T Cell-based immunotherapy in NSCLC treatment

### 4.1 Predictive value of T cell receptor dynamics in immunotherapy efficacy

As the central molecular determinant of T cell antigen recognition, TCR diversity metrics and clonal evolution are emerging as novel biomarkers for predicting immune checkpoint inhibitor efficacy. Han et al. (69) demonstrated in a seminal study that patients with high TCRβ chain CDR3 region diversity in peripheral PD-1^+^CD8^+^ T cells exhibited significantly superior disease control rates and survival benefits, with treatment-induced TCR clonal expansion correlating positively with tumor regression. The team further proposed the Tumor-Immune Repertoire (TIR) index—quantifying shared TCR clones between tumor and peripheral blood—showing that high TIR index patients achieved improved PFS and OS, mechanistically linked to elevated immunomodulatory cytokine levels (70). Notably, TCR clonal dynamics analysis effectively differentiates pseudoprogression. For instance, pseudoprogressive patients exhibit clonal expansion patterns and dominant clone overlap rates akin to partial responders, distinct from true progression cohorts, providing critical molecular insights for clinical decision-making. Zhang et al. (71) identified via multi-site TCR sequencing that only the top 1% high-frequency clones correlate with therapeutic response, with elevated tumor-peripheral TCR clonal concordance significantly enhancing major pathological response rates. These findings functionally complement prior CD4^+^/CD8^+^ T cell subset studies, collectively establishing a multidimensional predictive framework integrating T cell quantity and functional activity. Despite current limitations in sample sizes, TCR clonal monitoring demonstrates transformative potential in efficacy prediction, toxicity management, and progression discrimination, necessitating standardized sequencing protocols for clinical translation.

### 4.2 Radiotherapy-mediated remodeling of the tumor immune microenvironment

Radiotherapy remodels the TIME in NSCLC through multidimensional mechanisms, exhibiting dose-dependent biphasic immunomodulation. Preclinical studies demonstrate that conventional-dose radiotherapy activates PI3K/AKT and STAT3 signaling pathways to upregulate tumor cell PD-L1 expression while reducing immunosuppressive regulatory T cells (iTregs) and myeloid-derived suppressor cell (MDSC) infiltration, thereby promoting CD8^+^ T cell clonal expansion to establish an immunologically active niche (72). Preclinical models confirm synergistic antitumor effects when combining radiotherapy with PD-1/PD-L1 inhibitors, mechanistically linked to enhanced TCR diversity and spatial CD8^+^ T cell infiltration remodeling (73, 74). Notably, radiation fractionation patterns critically dictate immunomodulatory outcomes: hypofractionated radiotherapy outperforms conventional fractionation in activating systemic antitumor immunity via immunogenic cell death induction and proinflammatory cytokine release (75). A prospective cohort study by Theelen et al. (76) revealed that early-phase immune checkpoint inhibitor co-administration during radiotherapy synchronizes CD8^+^ T cell expansion peaks with radiation cycles, significantly improving objective response rates, though radiation pneumonitis risks require further evaluation. Current evidence highlights that spatiotemporal synergy between radiotherapy and ICIs transcends conventional therapeutic paradigms by reprogramming TIME immunoediting equilibria, offering advanced NSCLC patients a dual strategy for local control and systemic efficacy. Optimal intervention timing and safety management warrant validation through multicenter phase III trials.

### 4.3 Immunotherapy in non-small cell lung cancer

The therapeutic paradigm for non-small cell lung cancer has evolved from chemotherapy and radiotherapy to targeted therapies and, most recently, immune checkpoint inhibitors, which reprogram antitumor immunity by reversing T cell functional suppression. PD-1, a pivotal inhibitory receptor on T cells, initiates downstream immunosuppressive signaling upon interaction with tumor-expressed PD-L1/L2 ligands, driving T cell exhaustion and immune evasion (77, 78). ICIs restore T cell cytotoxic function by blocking the PD-1/PD-L1 axis while reactivating clonal expansion capabilities of tumor-infiltrating CD8^+^ T cells, thereby re-establishing antitumor immune surveillance networks (79, 80). Globally, multiple PD-1/PD-L1 inhibitors have been approved for NSCLC treatment, with over 200 related agents in clinical trials demonstrating synergistic therapeutic potential in combination with chemoradiotherapy or targeted therapies (81, 82). This precision strategy focused on reversing immunosuppression represents a paradigm shift in NSCLC treatment, moving beyond single-target approaches to achieve comprehensive immunomodulation.

### 4.4 TLS-mature CD8^+^ T cells to durable ICI responses in NSCLC

The immunological heterogeneity of NSCLC manifests in the spatial distribution of immune cells across tumor cores, invasive margins, and TLS, with hierarchical compartmentalization of effector populations: T lymphocytes and macrophages dominate as primary immune effectors, while plasma cells, NK cells, and myeloid-derived suppressor cells exhibit limited representation (83–86). In addition to core tumor and invasive margin compartments, tertiary lymphoid structures have emerged as crucial immunological hubs influencing NSCLC immunotherapy outcomes. These ectopic lymphoid aggregates, composed of B cells, T cells, follicular dendritic cells, and high endothelial venules, support local antigen presentation and clonal expansion. This profoundly immunosuppressive microenvironment subverts antitumor immunity through multifaceted mechanisms—defective antigen presentation impairs immune recognition, aberrant recruitment of Tregs establishes immune-tolerant niches, and sustained inhibitory cytokine networks suppress CD8^+^ T cell functional activity (12, 87). Recent studies have shown that TLS presence—particularly those containing mature CD8^+^ T cells—correlates strongly with durable responses to immune checkpoint inhibitor and improved overall survival. Spatial transcriptomic profiling confirmed that TLS-rich tumors exhibit enhanced infiltration of stem-like TCF1^+^CD8^+^ T cells, sustaining antitumor activity during prolonged ICI exposure (88–90). Furthermore, TLS density and maturation status may stratify patients beyond PD-L1 expression, offering a reproducible and spatially resolved biomarker for precision immunotherapy (91, 92). Integrating TLS profiling into prognostic models may substantially improve patient selection, therapeutic monitoring, and understanding of immune resistance dynamics (93).

## 5 Conclusion

Non-small cell lung cancer represents a paradigm of immune heterogeneity. The spatial distribution, phenotypic diversity, and functional states of tumor-infiltrating T lymphocytes critically determine disease progression and immunotherapeutic outcomes. This review highlights how distinct T cell subsets, particularly CD8^+^ cytotoxic T lymphocytes and Foxp3^+^ regulatory T cells, exert opposing immunological influences that shape the tumor immune microenvironment. The prognostic and predictive utility of these subsets depends not only on their density but also on their exhaustion status, clonal diversity, and localization within tumor compartments. Incorporating spatial transcriptomics, single-cell omics, and AI-assisted histopathological tools offers novel opportunities to refine TIME classification and advance immunotherapeutic precision.

Looking ahead, several actionable directions warrant attention. These include the development of standardized, spatially resolved biomarkers integrating TIL function and topography; dynamic monitoring frameworks that combine peripheral immune signatures with intratumoral exhaustion markers; personalization of immune checkpoint blockade through TCR repertoire analysis; and combinatorial strategies leveraging radiotherapy, ICIs, and adoptive T cell therapies to overcome resistance in non-inflamed tumor phenotypes. Multidimensional profiling and systems-level therapeutic design will be essential to transform NSCLC immunotherapy into a more precise, effective, and patient-tailored modality.
